# University Students and Their Ability to Perform Self-Regulated Online Learning Under the COVID-19 Pandemic

**DOI:** 10.3389/fpsyg.2022.781715

**Published:** 2022-03-09

**Authors:** Blanka Klimova, Katarina Zamborova, Anna Cierniak-Emerych, Szymon Dziuba

**Affiliations:** ^1^Department of Applied Linguistics, Faculty of Informatics and Management, University of Hradec Králové, Hradec Králové, Czechia; ^2^Department of English Language, Faculty of Applied Languages, University of Economics in Bratislava, Bratislava, Slovakia; ^3^Faculty of Business and Management, Wrocław University of Economics and Business, Wrocław, Poland

**Keywords:** self-regulated learning, online learning, motivation, metacognition, meaningfulness, personal competences, higher education

## Abstract

The COVID-19 pandemic has affected all aspects of the educational system, including students’ learning styles, which are heavily dependent on self-regulated studying strategies and motivation. The purpose of this study was to discover whether Central European students, in this case the Slovak and Czech students, were able to perform self-regulated learning during online learning under the COVID-19 pandemic to achieve their learning goals and improve academic performance, as well as to propose a few practical recommendations how to develop and maintain students’ self-regulation learning in this new online environment. The methodology was based on a questionnaire survey conducted among 268 students at two Central European universities in February and March 2021. The findings indicate that Central European students seemed to be able to perform their online self-study, especially in regard to personal competencies, meaningfulness and motivation. They reported higher awareness of their strengths and weaknesses in learning, time management, and/or the usefulness of making an effort to study. However, the findings reveal an urgent need for more work to be done in the area of metacognitive strategies, such as reflective and critical thinking, analyzing and evaluating. In this respect, the teacher’s role is replaceable since s/he serves as a facilitator and promotes these metacognitive strategies by providing students with constructive feedback, monitoring their learning, reviewing their progress, and/or providing opportunities to reflect on their learning. There were not any striking differences between the Czech and Slovak students. Nevertheless, Slovak students (females in particular) seemed to be more self-disciplined and goal-oriented in their learning.

## Introduction

The COVID-19 pandemic has brought changes for university students. Face-to-face teaching has been replaced by remote teaching with students getting used to a new academic environment. Students had to suddenly transit to more independent learning and self-study (Stradiotova et al., 2021; Zamborová et al., 2021). Self-regulated learning can be defined as one’s ability to understand and control one’s learning environment (Schraw et al., 2006). Kisac and Budak (2014) contend that self-regulation is proceeded by setting appropriate goals, selecting an effective learning approach, and monitoring progress toward these goals. As Paris and Paris (2001) put it, effective learners self-regulate, analyze task requirements, set productive goals, and select, adapt or invent strategies to achieve their objectives.

Exploration of self-regulation in learning has been a concept that still attracts the attention of a vast number of scholars worldwide (Hertel and Karlen, 2020; Jivet et al., 2020; Kryshko et al., 2020; Miná et al., 2021) and is applicable during these turbulent changes in education due to the pandemic. Particularly in the COVID-19 pandemic students have to do a lot of self-study, which requires much effort, self-determination and motivation on students’ side. And if they are not able to do this, they fail. Therefore, this study wants to explore whether students are able to conduct this self-study under new, challenging conditions, as well as discover whether there are differences between the students entering the university and those who have been there for some time already. The reason is that it is especially significant to introduce it to first-year students in higher education who are exposed to various challenges when entering a university. As a result, the dropout rate (e.g., 21% in Netherlands in 2016) is higher in the first year than in later years (Fokkens-Bruinsma et al., 2020). The reason lies in the importance of the foundation of knowledge and strategies in the first and later years. Especially attention needs to be paid to a smooth transition from secondary institutions to higher education. It concerns challenges in new students’ educational environments, new academic tasks, networking, acquiring a new identity, and competitiveness among peers (Fokkens-Bruinsma et al., 2020). Therefore, students need to be prepared for the expectations of studying at a university in the preparation phase, which currently has a lack of research (Fokkens-Bruinsma et al., 2020). Research conducted among first-year students concludes that time management and autonomous motivation are favorable predictors of achievement, while classroom engagement seems important later on. Students should have personalized trajectories from the moment they enter a university (Fokkens-Bruinsma et al., 2020). Thus, the authors of this study also want to propose a few practical recommendations how to develop and maintain students’ self-regulation learning in a new online environment to help them achieve their learning goals and successfully complete their university studies.

Self-regulation is comprised of four strategies/concepts that this article examines in the research section: motivation, personal competence, metacognitive strategies, and meaningfulness of learning. Recent research findings demonstrate that motivational regulation strategies increase students’ academic effort, their academic performance and reduce dropout intention (Kryshko et al., 2020). Motivation plays a role in the facilitation of learning and is connected to the integrity and quality of learning. Thus, *students with high motivation for success are usually successful students* (Ekşi et al., 2020). The research suggests that *there is a positive significant correlation between motivation for success and personal professional competence as well as a positive significant relationship between lifelong learning and personal-professional competency* (Ekşi et al., 2020). Students become intrinsically motivated if their psychological needs of autonomy, competence, and relatedness (based on the premise of self-determination theory) are satisfied in the academic context (Hensley et al., 2020). It is important to underline the fact that competence is the perception of being capable (Hensley et al., 2020). If the environment supports competence, students feel more confident in performing learning tasks and the teacher’s role is to support it. Therefore, students will not withdraw from trying (Hensley et al., 2020). Furthermore, personal competence includes several layers: cognitive, metacognitive, motivational, social/emotional, learning habits, mastery, enhancement, reinforcement, and contexts [see more in Redding (2014)]. The research by Hensley et al. (2020) showed evidence that to develop competence, there is a need for instructor scaffolding, required effort and analysis, and layers of meaning. Consequently, to reach a high level of self-regulation, proper **metacognitive strategies** should be utilized (Kisac and Budak, 2014). They refer to conscious monitoring, control learning, higher-order executive skills, and decision-making (Mitsea and Drigas, 2019). Their implementation encourages higher-order cognitive abilities, attentional and memory control, and self-confidence leading to independent and meaningful learning (Mitsea and Drigas, 2019). Kisac and Budak’s (2014) study investigated whether there was a link between university students’ metacognitive strategies and their perceived self-confidence levels about learning. They found that students who had higher self-confidence were more in favor of strategies like notetaking, summarizing, reflecting, reciting, and reviewing what they learned to things they had already known. It has been demonstrated that students with using effective metacognitive strategies can learn easily and effectively and have higher motivation and more self-confidence. Additionally, research by Hayat and Shateri (2019) showed that students with a strong belief about their ability to learn and complete the assignments are more effective in meeting requirements than students who are more skeptical. The last concept of self-regulated learning is **meaningfulness of learning**. One of the first studies by Nehari and Bender (1978) confirms that the meaningfulness and value of a course as judged by students are important factors in the cognitive-subject matter, affective-personal, and behavioral domains. Meaningfulness was explored in a study of 118 first year graduate social work students in the United States regarding the relationship among life satisfaction, peer support, and meaningfulness of the learning experience in connection to differences in gender, marital status, stress, and peer and family support. The study concluded that *receiving higher peer support was associated with perceived meaningfulness of the learning experience, whereas being female, being married, having lower perceived stress, and receiving higher family support were associated with life satisfaction* (Fakunmoju et al., 2016). To define meaning is to relate it to different ends beyond pleasure and the satisfaction of biological and material needs. Finding meaningfulness goes hand in hand with activities students consider worth pursuing, which leads to creating meaning for the entire life (Reber, 2018).

As mentioned, the COVID-19 outbreak has had an impact on the self-regulation learning practices of university students worldwide. As study in Spain evaluated how confinement at the beginning of pandemic affected the self-regulation of motivation (SRM) of university students and that the SRM was decreased by the shift from in-class teaching to virtual, and females outperformed males, although both genders showed SRM level reduction (Santamaría-Vázquez et al., 2021). Furthermore, a survey of college English learners’ self-regulation in an online environment in a Presentation-Assimilation-Discussion (PAD) class in China was carried out to examined self-preparation, self-management and self-evaluation. It was found that more colleges were well prepared for online English learning, and students had the ability to handle online learning (self-management) with suitable goals, plans, and most importantly, a good mood for learning. Their scores in self-evaluation stemmed from determination, right choice of strategies, reasoning through their learning progress, and adaptation to the PAD class (Zhenhua and Yanping, 2020). Interestingly, the study by Mayda et al. (2020) that examined the self-regulated learning skills of 209 sport science students in an online learning environment showed findings about females being more successful than males.

In the whole process of self-regulation learning, the role of a teacher is paramount (Oates, 2019). The findings of research studies (Boori and Ghanizadeh, 2011; Partovi and Tafazoli, 2016) show that it is especially emotional intelligence, self-efficacy, self-regulation, and critical thinking, which should be promoted by teacher. As Boori and Ghanizadeh (2011) state, the teacher should be equipped with them and model it to the students. Those are, for example, *goal setting, intrinsic interest, performance goal orientation, mastery goal orientation, self-instruction, emotional control, self-evaluation, self-reaction, and help seeking* (see Yesim et al., 2009). Oates (2019) expands that the teacher should model self-regulatory practices to stimulate students’ motivation for their own learning by engaging them in collaborative work and interventions. Naturally, there are other factors, which can also support the whole process of self-regulated learning, such as students’ abilities and willingness to engage in self-regulated learning, classroom environment, resources, curriculum, home and family background, parents, culture, and community (Alvi and Gillies, 2020).

Therefore, this study aimed to discover whether Central European students, in this case Slovak and Czech students, were able to perform self-regulated learning during their online classes in the period of the COVID-19 pandemic to achieve their learning goals and improve academic performance. In addition, the authors of this study want to discover whether there are any differences between these students as far as the year of study is concerned, gender or nationality. Finally, they also want to propose a few practical recommendations how to develop and maintain students’ self-regulation learning in a new online environment.

The research questions were as follows:

(1) *Were Czech and Slovak students able to perform self-regulated online learning under COVID-19 pandemic in order to achieve their learning goals?*
(2) *Which of the four self-regulation factors, i.e., motivation, personal competences, metacognition, and meaningfulness of studying, appeared to be most problematic?*
(3) *Were there any differences between the Czech and Slovak students?*
(4) *How can teachers support students’ self-regulation learning in a new online environment?*

## Methodology

### Participants

The research was performed in February and March 2021 at two Central European universities: one located in the Czechia (i.e., Faculty of Informatics and Management of the University of Hradec Králové) and one in Slovakia (Faculty of Applied Languages of the University of Economics in Bratislava). Both universities were of similar size. At the time of the survey, both groups of students had experience with self-study under the COVID-19 pandemic, since the previous semester was fully online as well. The survey was collected from 268 university students from the Czechia (*N* = 139) and Slovakia (*N* = 129) in their specialized courses of English as a foreign language. The research sample included students of economics and management, economic informatics, national economy, applied informatics, information management, and management of tourism. Their age predominantly ranged from 19 to 23 years.

### Instruments

The research instrument was a questionnaire developed by Hrbáčková (2011) containing demographic data (i.e., age, gender, year of study, form of study, and subject studied) and a list of 40 statements rated on a scale from 1 to 7 (1 denoting the least agree with the statement; 7 denoting most agree with the statement). All 40 statements are presented in four categories (motivation – 8 statements; personal competence – 16 statements; metacognition – 8 statements, and meaningfulness of learning – 8 statements).

Principal component analysis (PCA) was performed to reduce the 40 statements into fewer categories. Then the factors highlighted were used in further analysis of the approach to learning.

1. Verification of the PCA assumptions:
  a. KMO measure of sampling adequacy: **0.897** – this value means that reducing the number of categories makes sense.
  b. Bartlett’s test of sphericity: **4258.074 (*p* < 0.001)** – this value means the variables are correlated and, therefore PCA is justified.
2. Assumption of the number of extracted factors:

The following criteria were verified to extract a meaningful number of factors:

a. Sufficient proportion criterion: it assumes that the value of the cumulative percentage of the explained variance of the analyzed variables should be at least 75%. This would mean a reduction to **17 factors**.
b. Kaiser criterion: it implies the inclusion of the components that have eigenvalues higher than 1.0. This would mean a reduction to **8 factors**.
c. Cattell criterion: it is based on finding the factor scree, i.e., the location showing a gentle decrease of eigenvalues in a plot of eigenvalues grouped as non-descending. This means a reduction to **4 factors**.

**FIGURE 1:**
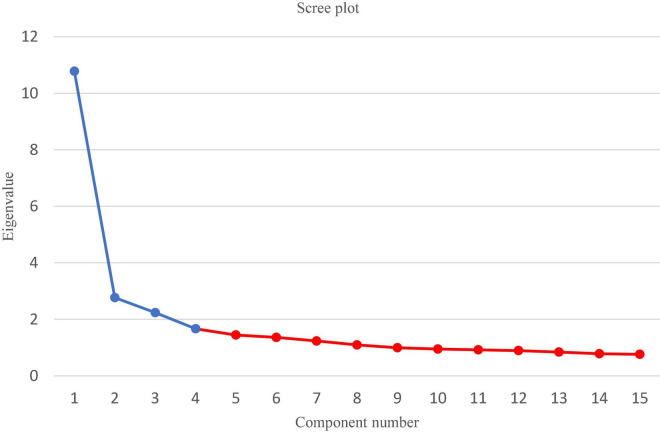
Reduction of factors: Cattell criterion. The red part shows that “gentle” decrease begins and at what point the reduction to a higher number (5 and more) of factors is pointless.

Although the Kaiser criterion is recommended for 20 or more variables, it was decided to adopt the Cattell criterion (Figure 1). This decision was dictated by the easier interpretation of the 4-factor model. Furthermore, the fact of the original classification of the statements into four thematic groups was taken into account.

#### Analysis of Factor Loading Matrix After Rotation

To classify the variables and obtain a clear arrangement of the loadings, the factor structure was rotated and the results were interpreted. The factor loading analysis allowed the creation of four scales, which were given the symbols C1, C2, C3, and C4. The distribution of the items is almost in accordance with the original structure of the survey questionnaire; therefore, the names of the factors were given according to the titles of the thematic groups:

• **C1** – *Motivation orientation*,
• **C2** – *Personal competence*,
• **C3** – *Metacognitive strategies*,
• **C4** – *Meaningfulness of studying.*

### Data Collection

When collecting data, all participants expressed agreement to participate in the online survey by taking part in it. It was fully voluntary and no instruction was given to them by the researchers. All GDPR regulations were strictly followed. The only demographic data we collected are presented in this manuscript without any personal identification. This research was approved by the Ethics Committee no. 2/2021 of the University of Hradec Králové.

## Results

The database to be statistically analyzed consisted of 139 records of Czech nationality students and 129 records of Slovak nationality students. In the next step, it was decided to aggregate the following data:

• **Age:** the raw database contains information about each respondent’s age without any division into ranges. For readability of the data interpretation, it was decided to aggregate the data into five ranges:
  • **A1** – respondents aged 17–19 years,
  • **A2** – respondents aged 20 years,
  • **A3** – respondents aged 21 years,
  • **A4** – respondents aged 22 years,
  • **A5** – respondents aged 23–36 years.
• **Year of study:** the raw database contained information about the year of study. Due to the low number of the respondents in their third, fourth, and fifth years of study, it was decided to combine the respondents into one group, i.e.,
  • **Y1 –** respondents in their first year of study,
  • **Y2 –** respondents in their second year of study,
  • **Y3 –** respondents in their third or higher years of study.

### Characteristics of the Respondents

The results in Table 1 show there were almost twice as many women as men in the Slovak group, while men outnumbered women among the Czechs. This is not surprising since students at the Faculty of Informatics and Management are predominantly males involved in computer science. However, eventually, the whole sample was characterized by a slight advantage of women (53.7 to 43.3%). The respondents were a mean age of less than 21 years and were most often in their first or second years of study (44.4 and 36.6%, respectively). Almost four-fifths of the Slovak group were first-year students, which was directly related to a lower mean age (20.2 years). Slightly more than half of the Czech students declared the second year of study, and this group was also older (mean age 21.6).

**TABLE 1:** Characteristics of the respondents.

Nationality	Personal data	Number of indications (N)	% Distribution	% Distribution by country
Czechia (CZ)		139	51.9%	
Slovakia (SK)		129	48.1%	
**Sex**				
CZ	Woman	59	22.0%	42.4%
	Man	80	29.9%	57.6%
SK	Woman	85	31.7%	65.9%
	Man	44	16.4%	34.1%
**Age**				
CZ	A1	4	1.5%	2.9%
	A2	24	9.0%	17.3%
	A3	44	16.4%	31.7%
	A4	37	13.8%	26.6%
	A5	30	11.2%	21.6%
SK	A1	30	11.2%	23.3%
	A2	63	23.5%	48.8%
	A3	28	10.4%	21.7%
	A4	5	1.9%	3.9%
	A5	3	1.1%	2.3%
**Year of study**				
CZ	Y1	17	6.3%	12.2%
	Y2	72	26.9%	51.8%
	Y3	50	18.7%	36.0%
SK	Y1	102	38.1%	79.1%
	Y2	26	9.7%	20.2%
	Y3	1	0.4%	0.8%

*Source: authors’ own study.*

### Analysis of the Results by Using Descriptive Statistics

The following table provides descriptive statistics for individual statements concerning the approaches to the self-regulated learning process. To ensure the readability of interpretation of the results, the scores for S1, S2, and S23 were reversed, as these statements, unlike the others, had negative overtones. The respondents agreed most with the statement “I know my strengths and weaknesses in learning” (total mean: 5.76), while the sentences with which they agreed least were “I buy or borrow additional recommended literature because I want to understand the field more” and “On my own initiative, I read the supplemental literature although it is not mandatory” (total means of 2.76 and 2.77, respectively). In addition, Table 2 below illustrates five statements with the largest differences between both group of students.

**TABLE 2:** Descriptive statistics of items included in the research questionnaire by nationalities.

Statement	Country	*M*	SD	MED	MOD
S1. I have to force myself to learn	CZ	3.39	1.54	3	4
	SK	3.81	1.45	4	3
S2. It often happens that I think of other things while learning	CZ	2.82	1.47	2	2
	SK	3.30	1.63	3	2
S3. I study even though I do not have to	CZ	3.12	1.64	3	2
	SK	3.51	1.63	4	2
S4. While studying, I fulfill the obligations beyond the requirements set by the teachers	CZ	3.63	1.64	4	2
	SK	4.02	1.53	4	4
**S5. On my own initiative, I read the supplemental literature although it is not mandatory**	**CZ**	**2.41**	**1.49**	**2**	**1**
	**SK**	**3.16**	**1.89**	**3**	**2**
S6. I like learning	CZ	4.41	1.50	4	4
	SK	4.46	1.58	5	5
S7. I buy or borrow additional recommended literature because I want to understand the field more	CZ	2.59	1.51	2	1
	SK	2.94	1.75	2	1
S8. I read study materials (notes from lectures, university textbooks, etc.) on an ongoing basis	CZ	4.18	1.71	4	5
	SK	4.18	1.57	4	4 and 5
S9. I can estimate the demands placed on me during my studies	CZ	4.52	1.30	5	4
	SK	4.80	1.16	5	4
S10. I know which style of learning is most appropriate in a given situation	CZ	4.61	1.41	5	5
	SK	4.98	1.37	5	5
S11. I know my strengths and weaknesses in learning	CZ	5.81	1.13	6	6
	SK	5.77	1.22	6	6
**S12. I can organize my study materials so I can study them well**	**CZ**	**5.00**	**1.43**	**5**	**5**
	**SK**	**5.50**	**1.44**	**6**	**7**
S13. I expect to do well in my studies	CZ	4.95	1.37	5	6
	SK	5.24	1.21	5	5
S14. I have my studies under control and I know how well I understand the issues studied	CZ	4.72	1.29	5	5
	SK	4.80	1.35	5	5
S15. I do not give up easily, even I do not understand something	CZ	5.09	1.32	5	6
	SK	5.28	1.27	5	6
S16. I know what information is the most important and which is of less importance	CZ	4.95	1.28	5	5
	SK	4.96	1.16	5	5
S17. I have a good memory	CZ	4.50	1.53	5	5
	SK	4.84	1.42	5	5
S18. I believe when I decide to succeed, I can	CZ	5.45	1.39	6	6
	SK	5.50	1.29	6	7
S19. I can organize my time to learning in a way to succeed on exams	CZ	5.05	1.45	5	6
	SK	5.23	1.25	5	5
S20. If I know what makes it difficult for me to learn, I can resolve or easy the challenge	CZ	4.27	1.19	4	4
	SK	4.55	1.21	4	4
S21. I am not afraid to start with the more demanding tasks needed to complete what I am studying	CZ	4.31	1.43	4	4 and 5
	SK	4.52	1.36	4	4
S22. I think I am better at studying than most of my classmates	CZ	3.43	1.42	3	3
	SK	3.19	1.37	3	4 and 3
S23. I often have a feeling that I don’t understand anything and won’t master the study	CZ	4.11	1.71	4	4
	SK	4.58	1.68	5	6
S24. The moment I complete the test, I know I passed it successfully	CZ	3.94	1.33	4	4
	SK	3.81	1.53	4	4
S25. When learning, I need to make sure I am moving in the right direction	CZ	4.75	1.40	5	6
	SK	4.94	1.33	5	5
S26. I often find myself stopping while learning to check that I understand everything	CZ	4.27	1.40	4	5
	SK	4.68	1.53	5	4
S27. When learning new information, I ask myself questions to find out how I am doing	CZ	3.81	1.65	4	3
	SK	3.82	1.68	4	4
S28. Before I start learning, I describe what I will do to myself (now and later)	CZ	3.66	1.88	4	3
	SK	3.98	1.81	4	3
**S29. When I am learning, I constantly test myself to see if I have really understood the subject matter**	**CZ**	**3.92**	**1.49**	**4**	**4**
	**SK**	**4.56**	**1.52**	**5**	**5**
**S30. I often ask myself if I have done everything I can to understand the subject**	**CZ**	**3.62**	**1.59**	**4**	**3**
	**SK**	**4.39**	**1.48**	**5**	**5**
S31. It often happens that when I am learning, I analyze or evaluate myself	CZ	3.72	1.52	4	4
	SK	3.99	1.50	4	4
**S32. When learning, I usually divide the learning materials into several parts, which I learn gradually**	**CZ**	**4.56**	**1.87**	**5**	**5**
	**SK**	**5.19**	**1.59**	**5**	**6**
S33. I try to relate the information I learn in one subject to other subjects	CZ	5.00	1.51	5	5
	SK	5.09	1.42	5	5
S34. I like the content of subjects studied in this field	CZ	4.69	1.40	5	5
	SK	4.91	1.31	5	6
S35. I think it is useful to make effort to study	CZ	5.49	1.37	6	5
	SK	5.31	1.31	5	5
S36. I am interested in the subjects studied in this field	CZ	4.91	1.44	5	5
	SK	4.98	1.34	5	6 and 5
S37. It is very important for me to understand the issues studied	CZ	4.96	1.41	5	5
	SK	5.29	1.36	5	5
S38. I learn by combining information from several sources (notes from lectures, university textbooks, recommended literature, etc.)	CZ	4.72	1.55	5	5
	SK	4.88	1.67	5	6
S39. I study as a hobby	CZ	3.12	1.76	3	2
	SK	3.22	1.88	3	1
S40. I think that what I am learning in my studies can be used in practice	CZ	4.92	1.54	5	6
	SK	5.22	1.43	5	5 and 6

*Source: authors’ own study. Bold letters indicate the largest differences between both groups of students.*

No statistically significant differences were found for most statements. Item numbers, Mann–Whitney *U*-statistics, and *p*-value for the statements in which significantly different responses were observed between Czechs and Slovaks are presented in Table 3. In all the statements, a higher average score was observed for Slovak students.

**TABLE 3:** Final classification of statements into scales and reliability of scales.

Scale	Items	Cronbach’s alpha	
		CZ	SK
Full scale	–	0.914	0.934
Motivation orientation	S1 S2	0.804	0.844
	S3 S4		
	S5 S6		
	S7 S8		
	S39		
Personal competence	S9 S10	0.860	0.885
	S11 S12		
	S13 S14		
	S15 S16		
	S17 S18		
	S19 S20		
	S21 S22		
	S23 S24		
Metacognitive strategies	S25 S26	0.795	0.774
	S27 S28		
	S29 S30		
	S31 S32		
Meaningfulness of studying	S33 S34	0.819	0.846
	S35 S36		
	S37 S38		
	S40		

*Source: authors’ own study.*

### Factor Analysis

Table 3 shows the final classification of the variables and the reliability coefficients of the scales for each nationality. The least consistent was the *Metacognitive strategies* scale, with a Cronbach’s alpha coefficient of 0.795 for Czech students and 0.774 for Slovak students. The best reliability was recorded for the *Personal competence* scale. In the Czech survey, it was 0.860, whereas in the Slovak survey, it was 0.885. No statement was excluded during the analysis (the values of the coefficients after removing the items were at most at the same level, but most often lower).

#### Analysis of Demographic Variables

As Table 4 illustrates, the Slovak students rated *Metacognitive strategies* (*p* < 0.001) and *Motivation orientation* (*p* = 0.016) significantly higher than the Czech students.

**TABLE 4:** An overview of the analysis by nationality.

Scale	CZ (*n* = 139)		SK (*n* = 129)		Significance of differences
	*M*	SD	*M*	SD	
Motivation orientation	3.3	1.0	3.6	1.1	*U* = 7441.5
					*p* = 0.016*
Personal competence	4.7	0.8	4.8	0.8	*U* = 8161.0
					*p* = 0.205
Metacognitive strategies	4.0	1.0	4.4	1.0	*U* = 6873.0
					*p* < 0.001***
Meaningfulness of studying	4.7	1.0	4.9	1.0	*U* = 8424.0
					*p* = 0.393

*Source: authors’ own study.*

The “*” symbol means that p-value is between 0.01 and 0.05.

The “**” symbol means that p-value is between 0.001 and 0.01.

The “***” symbol means that p-value is lower than 0.001.

No statistically significant differences were found in either women or men (Table 5).

**TABLE 5:** An overview of the analysis by age.

Scale	A1					A2					A3				
	CZ (*n* = 4)		SK (*n* = 30)		Significance of differences	CZ (*n* = 24)		SK (*n* = 63)		Significance of differences	CZ (*n* = 44)		SK (*n* = 28)		Significance of differences
	*M*	SD	*M*	SD		*M*	SD	*M*	SD		*M*	SD	*M*	SD	
Motivation orientation	3.7	0.7	3.6	1.1		3.4	1.0	3.6	1.0	*U* = 737.5	3.2	1.1	3.9	1.2	*U* = 408.0
										*p* = 0.860					*p* = 0.016*
Personal competence	4.4	0.8	5.1	0.6		4.7	0.8	4.8	0.9	*U* = 739.0	4.8	0.7	4.9	0.8	*U* = 600.5
										*p* = 0.872					*p* = 0.858
Metacognitive strategies	4.8	0.9	4.6	0.8		4.2	1.2	4.5	0.9	*U* = 678.5	4.1	1.1	4.4	1.2	*U* = 526.0
										*p* = 0.461					*p* = 0.298
Meaningfulness of studying	4.9	1.1	4.7	1.0		4.9	1.1	4.7	1.0	*U* = 638.0	4.8	1.0	4.9	1.1	*U* = 615.5
										*p* = 0.262					*p* = 0.995

*Source: authors’ own study.*

The “*” symbol means that p-value is between 0.01 and 0.05.

The “**” symbol means that p-value is between 0.001 and 0.01.

The “***” symbol means that p-value is lower than 0.001.

For the 1st, 4th, and 5th age groups, significance tests were not conducted due to the insufficient sample size. There was a correlation in the case of age group 3: students from Slovakia rated the approach to self-regulated learning significantly higher in terms of *Motivation orientation* (*p* = 0.016).

Students in their first year of study from Slovakia rated the *Personal competence* aspect higher than their peers from the Czechia. For students in their third and higher year of study, significance tests were not conducted due to the insufficient sample size of Slovak students (Table 6).

**TABLE 6:** An overview of the analysis by year of study.

Scale	Y1					Y2					Y3				
	CZ (*n* = 17)		SK (*n* = 102)		Significance of differences	CZ (*n* = 72)		SK (*n* = 26)		Significance of differences	CZ (*n* = 50)		SK (*n* = 1)		Significance of differences
	*M*	SD	*M*	SD		*M*	SD	*M*	SD		*M*	SD	*M*	SD	
Motivation orientation	3.5	1.0	3.6	1.1	*U* = 838.5	3.5	1.0	3.9	1.1	*U* = 700.0	3.0	0.9	2.0	0.0	
					*p* = 0.829					*p* = 0.057					
Personal competence	4.2	0.8	4.9	0.8	*U* = 487.5	4.8	0.7	4.8	0.9	*U* = 875.0	4.6	0.8	4.1	0.0	
					*p* = 0.004**					*p* = 0.623					
Metacognitive strategies	4.1	1.3	4.5	0.9	*U* = 700.0	4.1	1.1	4.4	1.1	*U* = 774.0	3.9	0.9	5.4	0.0	
					*p* = 0.204					*p* = 0.192					
Meaningfulness of studying	4.7	1.1	4.9	1.0	*U* = 796.0	4.9	1.0	4.8	1.2	*U* = 868.0	4.5	1.0	3.8	0.0	
					*p* = 0.589					*p* = 0.586					

*Source: authors’ own study.*

The “*” symbol means that p-value is between 0.01 and 0.05.

The “**” symbol means that p-value is between 0.001 and 0.01.

The “***” symbol means that p-value is lower than 0.001.

## Discussion

The findings described above indicate that **Central European students seemed to be able to perform their online self-regulated study, especially as far as personal competence, meaningfulness and motivation are concerned**. And this is generally true regardless students’ gender, year of study and nationality. Overall, students reported higher awareness of their strengths and weaknesses in learning, time management, and/or usefulness of making an effort to study. In addition, they feel able to interconnect the knowledge of one subject with the knowledge of another subject, which confirms their awareness of interdisciplinary knowledge.

On the contrary, **they appeared to have problems with their metacognitive strategies**, although Slovak students did slightly better. This includes, for example, less reflecting on one’s own learning or analyzing what they are going to do next – skills necessary for higher-order cognitive skills (cf. Mitsea and Drigas, 2019). They are not well-prepared for self-study from their institutions of secondary school learning where they were more used to memorization and fewer discussions. The students were suddenly given a huge amount of literature and assignments to do their own, whereas there were still tendencies to be fully guided and checked by their teachers at their former schools. Therefore, there is an urgent need to start developing self-regulated behaviors for studying in the early years and accentuate its importance at the secondary institutions by encouraging students to do their own research, to prioritize their tasks, and to organize their time and ability to work with much information. Furthermore, the Slovak students seemed to be more motivated, particularly in their second year of study as they experienced the school load in their first year, got to know the teachers and formed social clubs in their study groups to share and exchange information and study materials. After the first-year experience, they could better organize their study time, assignments, and requirements so they could enjoy free-time activities. Ambitious students could take advantage of exchange study trips, and some might have found a part-time job in their area of studies so they had hands-on experience to utilize in their studies and careers after graduation. Nevertheless, overall, the findings of this study indicate that metacognitive strategies improve with higher motivation and personal professional competence, which was also confirmed by Kisac and Budak (2014). In addition, students in both countries perceived their studies as meaningful, such as usefulness of studying.

**There were not any striking differences between the Czech and Slovak students.** Nevertheless, Slovak students (females in particular) seemed to be more self-disciplined and goal-oriented in their learning. This result could have been also affected by a higher proportion of boys in the Czech sample. However, as shown in the PISA testing, Slovak females generally score higher than males. Similar findings about female students were also confirmed by Mayda et al. (2020) and Santamaría-Vázquez et al. (2021).

Although learning does not seem to be the students’ principal hobby, they thought it was useful to make effort to study. Both group of students seemed to be motivated by the competitive salaries possible in their fields of study. They take part-time jobs as their school load and schedule allow them to earn extra cash and get experience. They also realize that without the university diploma, the doors to the job market are closed.

Since there are abundant learning materials online, students prefer not to buy or lend any course materials, as the findings of this study revealed. They appear to be more ecologically friendly than their former peers. Even though there are enough information and resources online, students do take notes, highlight the most important information and learn the given material by heart.

Therefore, the **teacher should gradually introduce self-regulated strategies** from Day 1 so that students are aware of consciously working toward them. One needs to, however, realize that it is a life-long learning process but good basis can be grounded even within a semester of studying. Additionally, as the study by Partovi and Tafazoli (2016) reveals, the more experienced EFL teachers are, the more self-regulatory traits they have in altogether with more resilience in the students’ point of view. Therefore, teachers as a model in developing their own self-regulation with time might be an encouraging and decisive factor in students’ learning development.

On the basis of the findings of this study and with respect to the findings of other research studies (e.g., Alvi and Gillies, 2020), the following practical recommendation could be provided in order to successfully develop and maintain students’ self-regulated learning, especially their metacognitive strategies that they still lack at this level:

• Being at students’ disposal in a timely manner to keep them motivated in their studies, as well as guiding them in their online learning environment,
• Helping students link new experiences to prior learning by applying their newly acquired knowledge and skills in broader contexts and in the case of foreign language learning to expose them to authentic environment (e.g., involving native speakers),
• Providing students with corrective and timely feedback by focusing on the task of learning, not on the learner,
• Giving students opportunities to communicate with each other, not only the teacher in order to share their expertise and emotions about their learning,
• Promoting students’ reflection, for example, by writing self-reflection diaries or essays in a critical manner, and thus developing also their critical thinking,
• Adjust assessment criteria due to the new learning environment, as well as encourage students to keep a track of their own self-assessment rubrics which may help them find a gap in their new and past acquired knowledge,
• Put an emphasis on the new product outcome due technological innovations and needs of the current generation of students (e.g., record a video, create an interactive presentation, work with the mobile apps and softwares).

The limitations of this study consist of unbalanced gender samples, i.e., in the Slovak sample was predominantly females and the relatively small scale of the research, as it was conducted in only two neighboring European countries. Therefore, it would be necessary to replicate the research on a much larger scale to obtain more generalizable results. Nevertheless, this research generated statistically relevant and reliable results. Future research should focus on verification of our findings on a larger scale and in a larger geographical area.

## Conclusion

Generally, it can be concluded that the present pandemic students in Central Europe who had to study only online for the entire academic year seemed to be able to perform self-regulated online learning. However, the findings show that much more work must be done in developing their metacognitive strategies, such as reflective and critical thinking, analyzing or evaluating, the strategies that are crucial for successful academic performance. In this respect, it is the teacher who can serve as a facilitator and promote these metacognitive strategies among his/her students by providing students with constructive feedback, monitoring their learning, reviewing their progress, and/or providing opportunities to reflect on their learning.

## Data Availability Statement

The original contributions presented in the study are included in the article/supplementary material, further inquiries can be directed to the corresponding author.

## Ethics Statement

The studies involving human participants were reviewed and approved by the Ethics Committee no. 2/2021 of the University of Hradec Králové. The patients/participants provided their written informed consent to participate in this study.

## Author Contributions

BK, KZ, AC-E, and SD drafted, analyzed, wrote, and read the whole manuscript. All authors contributed to the article and approved the submitted version.

## Conflict of Interest

The authors declare that the research was conducted in the absence of any commercial or financial relationships that could be construed as a potential conflict of interest.

## Publisher’s Note

All claims expressed in this article are solely those of the authors and do not necessarily represent those of their affiliated organizations, or those of the publisher, the editors and the reviewers. Any product that may be evaluated in this article, or claim that may be made by its manufacturer, is not guaranteed or endorsed by the publisher.
